# Loneliness and cognitive function among Chinese older adults: multiple mediating effects of activities of daily living and anxiety

**DOI:** 10.3389/fpubh.2026.1887804

**Published:** 2026-06-25

**Authors:** Zheng Ren, Yujie Li, Wuniu Jida, Lele Xiao, Yu Wei

**Affiliations:** 1 School of Politics and Public Administration, Guangxi Normal University, Guilin, China; 2 Western Urban and Rural Integration Development Institute, Guilin, Guangxi, China; 3 Guilin Maternal and Child Health Hospital, Guilin, Guangxi, China

**Keywords:** activities of daily living, anxiety, cognition function, loneliness, older adults

## Abstract

**Background:**

Several studies have found that loneliness is a key factor affecting cognitive function. However, the underlying mechanism of activities of daily living (ADL) and anxiety in this effect have not been investigated. This study aims to explore the mediating effects of ADL and anxiety on the relationship between loneliness and cognitive function among Chinese older adults.

**Methods:**

In this study, 10,026 older people aged 60 and above were included based on the 2018 Chinese Longitudinal Healthy Longevity Survey (CLHLS). Cognitive function, anxiety, ADL, and loneliness were evaluated using the mini-mental state examination, the 7-item generalized anxiety disorder scale, the Katz index scale, and the single-item, respectively. The mediation analysis was then conducted using the PROCESS macro method and multiple regression analysis.

**Results:**

It was observed that the overall proportion of older adults with cognitive impairment was 25.3%. Loneliness, ADL, and anxiety showed significantly negative correlations with cognitive function, with a correlation coefficient of −0.144, −0.615 and −0.105, respectively. The indirect effects of ADL and anxiety on the relationship between loneliness and cognitive function were −0.1159 and −0.0708, respectively, which represented 30.0% and 18.3% of the total effect, respectively. The serial indirect effect of ADL and anxiety was −0.0008, accounting for only 0.2% of the total effect.

**Conclusion:**

This study demonstrated the necessity of addressing the status of cognitive impairment among Chinese older adults. ADL and anxiety may independently and accumulatively mediate the relationship between loneliness and cognitive function.

## 1 Introduction

The global population of older adults is expected to reach 1.4 billion by 2030. Such an aging trend is also anticipated to continue, with an increase to 2.1 billion by 2050 (1). With the acceleration of the global population aging process, cognitive impairment featured by the difficulty, loss or decline of cognitive capacities has become an important public health problem threatening the health of older adults (2). Over time, the severity of cognitive impairment progresses from mild cognitive impairment (MCI) to Alzheimer’s disease and dementia. According to the World Health Organization, it is expected that the number of people with dementia will reach 139 million by 2050 (3). The overall prevalence of MCI is determined to be 19.6%, and the crude prevalence of dementia is 9.1% in the Chinese population (4, 5). The burden of dementia has posed major social, financial, and public health challenges around the world. However, it is worrying that current anti-dementia medications provide only temporary symptomatic relief without preventing the underlying neurodegenerative progression. Therefore, proactive monitoring and early intervention targeting modifiable risk factors in the cognitive health of older adults may represent a pivotal opportunity to delay or even prevent the onset of debilitating dementia.

The impoverished social relationship (defined as social isolation or loneliness) is identified as a modifiable risk factor for poor outcomes of cognitive health in later life (6). Loneliness is a subjective emotional state derived from perceived differences between people’s expected and actual social relationships, both in quantity and quality (7). It has become the most common negative emotion among older adults. The longitudinal relationship between changes in loneliness and cognitive decline has been investigated in several Western-based studies, but the evidence remains inconclusive (8, 9). Cognitive reserve theory suggests that social connection can increase synaptic density and neurogenesis in the brain. Therefore, the greater the cognitive reserve of individuals, the lower the risk of neurodegenerative diseases (10, 11). In China, both chronic and transient loneliness are important predictors of cognitive decline in older adults (12).

Independent physical and cognitive abilities are important components of healthy life expectancy in older adults. Activities of daily living (ADL), as an indicator for measuring physical function, refers to a series of activities in daily life, including basic ADL (BADL) and instrumental ADL (IADL) (13). Globally, more than 1 billion people experience one or more forms of disability, which suggests that functional disability poses an important public health challenge (14). The assessment of ADL is crucial for diagnosing neurocognitive dysfunction, as both BADL and IADL limitations have been shown to predict cognitive impairment (15, 16). It is believed that higher levels of loneliness are related to increased self-reported ADL impairment (17). Activity theory of ageing highlights that active participation in physical and social activities is critical for promoting the well-being of older adults (18). Therefore, functional independence can serve as a bridge between loneliness and cognitive health outcomes. Loneliness, as a psychosocial stressor, may trigger social withdrawal and disengagement from daily activities, which weakens the ability of older adults to perform self-care and complex tasks. This in turn deprives them of the cognitive challenges necessary to maintain brain plasticity and delay neurodegeneration. Hence, the hypothesis that (H1) ADL can mediate the relationship between loneliness and cognitive function is proposed.

Anxiety symptoms are commonly characterized by persistent restlessness, excessive concern, or heightened apprehension, affecting millions of people worldwide, especially older adults. A systematic review and meta-analysis revealed that 28% of older individuals experienced significant anxiety symptoms (19). Anxiety is increasingly recognized as a potential risk factor for cognitive decline. Studies have demonstrated that the progression of the neurodegenerative spectrum from mild cognitive impairment to dementia is accelerated by anxiety (20, 21). Processing efficiency theory shows that anxiety interferes with cognitive performance by preemptively occupying working memory capacity and executive control systems, which impairs performance on attention-demanding tasks (22). Therefore, appropriate treatment of anxiety symptoms may have beneficial effects on the risk of neurodegenerative disease development. Loneliness is a painful experience that has been proven to be a stressor harmful to psychological, physical, and cognitive health (23). It has been also reported that loneliness is related to anxiety symptoms and causes enhanced stress responses (24, 25), which is related to long-term activation of the hypothalamic–pituitary–adrenal axis and the sympathetic-adrenal gland. These maladaptive physiological responses may foster neuroinflammation and hippocampal atrophy to culminate in cognitive impairment. Therefore, the hypothesis that (H2) anxiety may mediate the relationship between loneliness and cognitive function is proposed.

Functional independence is a critical determinant of mental health in older adults. Sustained social interactions are crucial for fostering psychological resilience and reducing emotional vulnerability to negative experiences. However, ADL limitations inevitably increase dependence on others and restrict social engagement, thereby elevating vulnerability to anxiety (26). A previous study linked ADL impairments to a higher probability of psychological distress (27). This connection between psychological distress and cognitive decline is theoretically reinforced by recent evidence. Rodrigues et al. (28) identify loneliness as a critical interface and comorbid factor bridging Alzheimer’s disease and suicidal behavior, highlighting a shared psychobiological pathway. Grounded in this framework, the anxiety triggered by ADL limitations can be viewed as a manifestation of this interface, potentially accelerating cognitive decline. Hence, the hypothesis is that (H3) loneliness is negatively related to cognitive function through the chained mediation pathway of ADL and anxiety.

**Figure 1:**
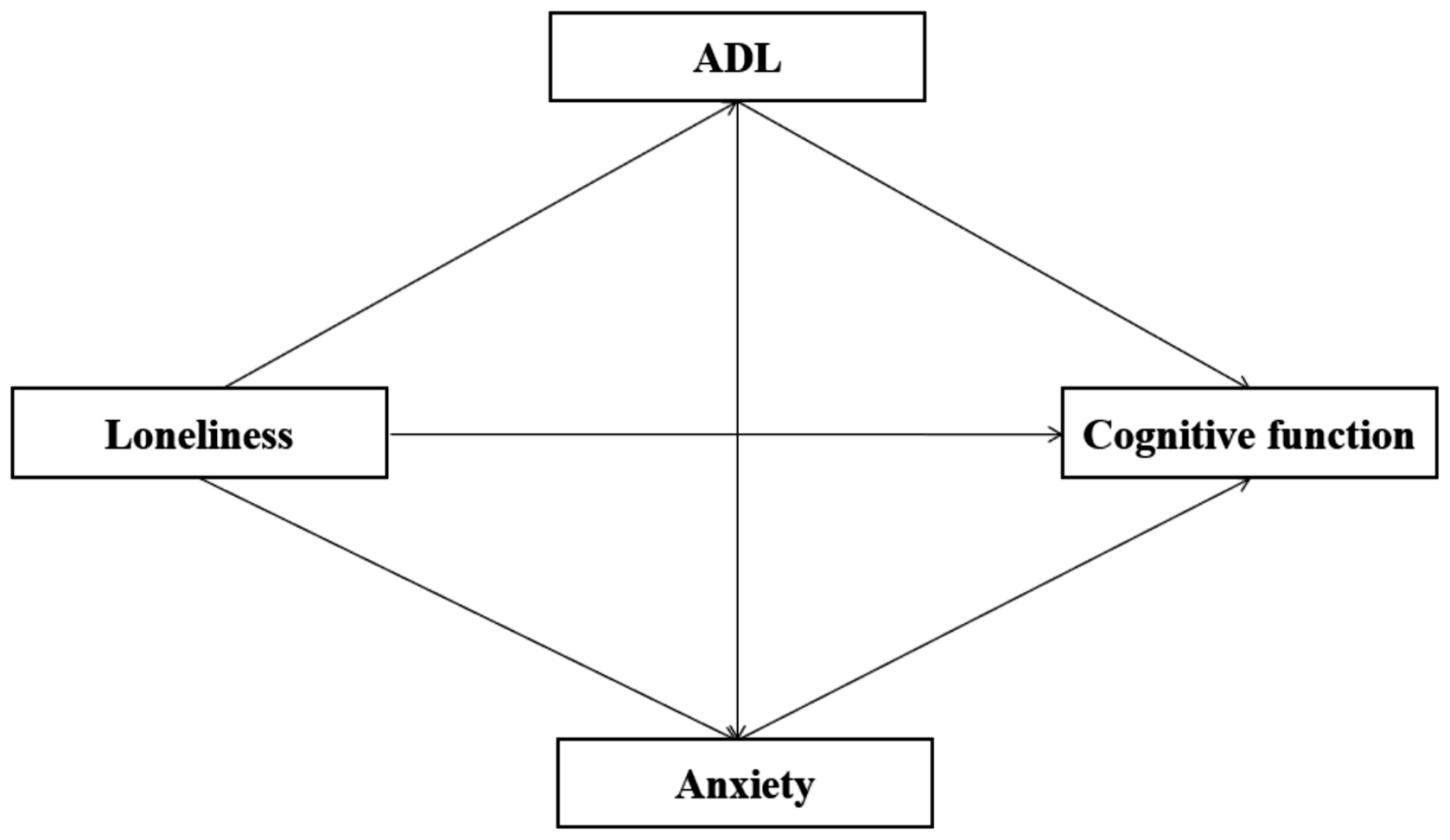
The mediating effects of ADL and anxiety on the association between loneliness and cognitive function.

The present study aims to characterize the status of cognitive function and elucidate the chained mediating roles of ADL and anxiety in the association between loneliness and cognitive function. Specifically, we propose that loneliness is negatively associated with cognitive function, with ADL (H1) and anxiety (H2) mediating this relationship independently, and that ADL and anxiety serve as serial mediators (H3). These findings will deepen our understanding of the underlying mechanisms linking loneliness to cognitive decline, offering valuable implications for developing targeted interventions against cognitive impairment. The proposed theoretical model is depicted in Figure 1.

## 2 Methods

### 2.1 Data source

**Figure 2:**
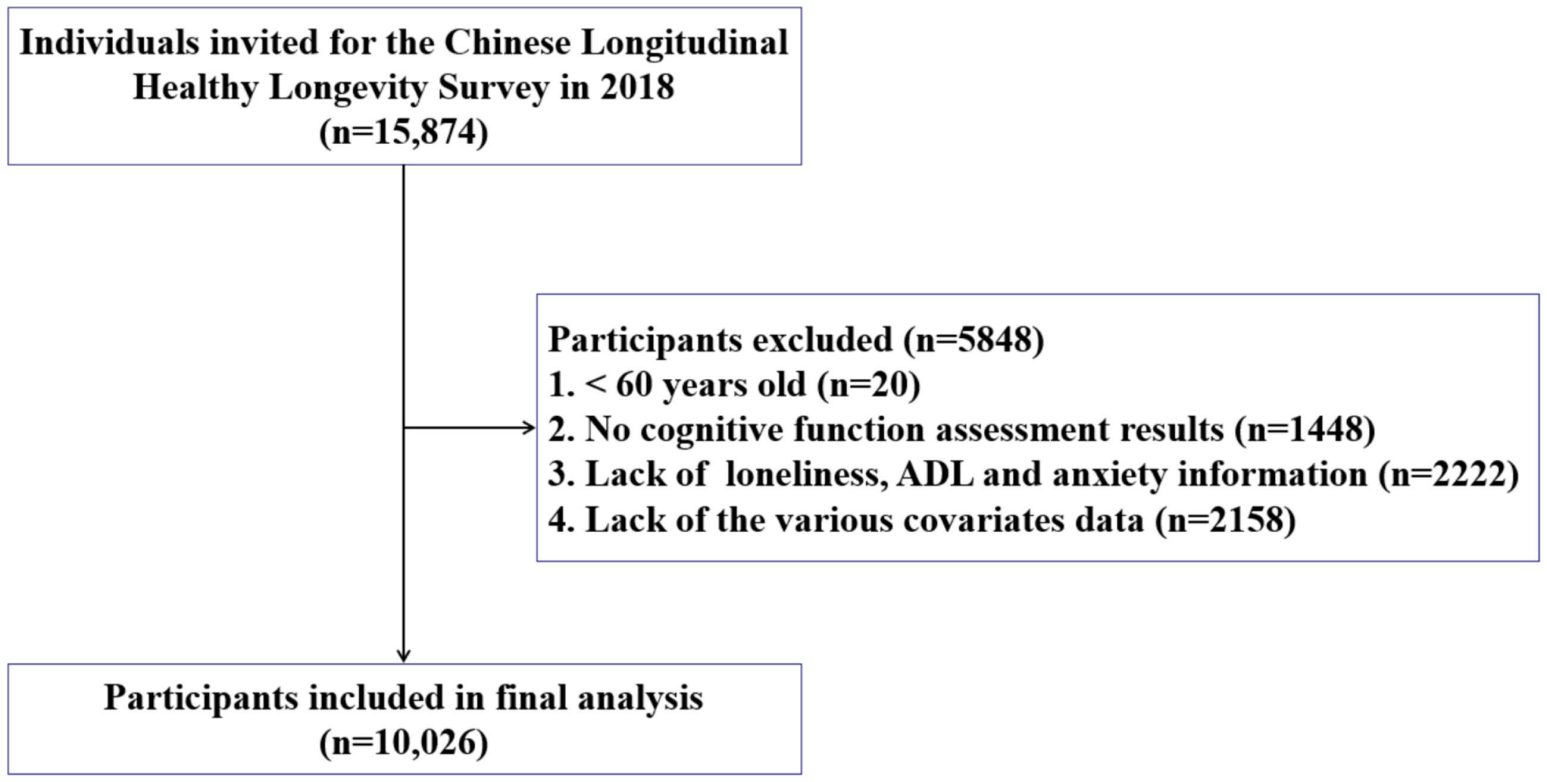
The research selection process of participants.

Data were obtained from the 2018 Chinese Longitudinal Healthy Longevity Survey (CLHLS) which is a longitudinal and international cooperation project. The first wave of data was collected in 1998, which interviewed older adults aged 80 and over from 22 provinces in China. Seven waves of follow-up were performed in 2000, 2002, 2005, 2008/2009, 2011/2012, 2014, and 2018. Data were systematically collected from CLHLS through face-to-face interviews, with generally good quality (29). In the 2018 wave, 15,854 participants aged 60 and above were surveyed, excluding 3,670 participants due to missing data on key variables such as loneliness, ADL, anxiety, and cognitive function. Also, 2,158 participants who did not complete the information about covariates were excluded. Finally, a total of 10,026 older adults aged 60 and above were included in this study. The full process of the inclusion and exclusion of respondents is shown in Figure 2. This study was carried out according to the guidelines stipulated in the Declaration of Helsinki, and the data already obtained the ethical approval and informed consent, and was approved by research ethics committees of Duke University and Peking University (IRB00001052-13074). As a secondary analysis of existing data, no *a priori* sample size calculation was conducted. The final sample (*n* = 10,026) included all eligible participants, and the effect sizes are presented in the mediation analysis.

### 2.2 Measures

#### 2.2.1 Loneliness

Loneliness was used as the independent variable of this study. The single-item survey method for assessing loneliness has been shown to be effective and highly associated with multiple loneliness scales (30, 31). In this survey, loneliness was measured by asking how often the respondent felt lonely. The response was scored by a range of 1–5, with 1 for “always” and 5 for “never.” Thus, higher scores indicate lower loneliness in older adults. The score is reverse-coded, which means that higher scores indicate greater feelings of loneliness. On this basis, a score of <3 is expressed as “not lonely” and a score of ≥3 is expressed as “lonely” (32). To show the level of loneliness, “sometimes,” “often” and “always” were grouped into one category, while “seldom” and “never” were grouped into another category.

#### 2.2.2 ADL

Activities of daily living was used as one mediator variables of this study. The total score of BADL and IADL was used for the comprehensive evaluation of ADL. BADL was assessed by the Katz index scale (33), which consists of six items: bathing, dressing, toileting, indoor activities, continence, and eating. Each answer was divided into three responses: independent, needing help, or dependent. In this study, the original answer for each BADL item was recorded as 0 if the respondents reported that they completed the activity independently, 1 if the respondents reported that they needed help to complete the activity, and 2 if the respondents reported that they completed the activity by depending on others. BADL disability was defined as one of the six elements that cannot be done alone. IADL was assessed by the modified Lawton’s scale (34), which consists of eight items: cooking, shopping, washing clothes, visiting neighbors, taking public transportation independently, lifting a weight of 5 kg, walking for 1 km, and crouching and standing for three times continuously. The response classification and coding of IADL were similar to those of BADL. The total score of ADL ranged from 0 to 28, with higher scores indicating worse ADL. In this study, the Cronbach’s alpha coefficients of Katz index scale and Lawton’s scale were 0.858 and 0.949, respectively.

#### 2.2.3 Anxiety

Anxiety was used as another mediator variable. The 7-item Generalized Anxiety Disorder scale (GAD-7) was used to evaluate anxiety symptoms in older adults (35), which has been validated in older Chinese (36). Participants aged 60 and above were asked to recall a series of feelings from the last 2 weeks, such as “feeling restless, anxious and annoyed,” “unable to stop or control anxiety,” and “worrying too much about everything.” There were four options for each item: 0 = never, 1 = for several days, 2 = more than half a day, 3 = almost every day. The total score ranged from 0 to 21, and higher scores indicated more severe anxiety. A score greater than or equal to 5 was considered as the cut-off point for classifying the status of anxiety (37). In the current study, the Cronbach’s alpha coefficient of this scale was 0.917.

#### 2.2.4 Cognitive function

Cognitive function as an outcome variable was assessed using the 24-item Chinese version of the Mini-Mental State Examination (MMSE). The MMSE questionnaire assessed language skills, recall, drawing, calculation, attention, one-minute food counting, and orientation. The question “Say the number of food items in 1 min” had a maximum score of 7 points. The other 23 items had a maximum of 1 point each. The MMSE score ranged from 0 to 30 and higher scores indicated better cognitive function. Participants with a MMSE score below 24 were defined as having cognitive impairment (38). The validity and reliability of the Chinese MMSE have been verified in a previous study (39). In this study, the Cronbach’s alpha values of MMSE were 0.890.

#### 2.2.5 Covariates

In this study, covariates were included and defined as follows: Sociodemographic variables including gender (male or female), age (year), marriage status (have a spouse or not), educational level (illiterate or non-illiterate), place of residence (city and town, rural), and co-residence status (living in nursing home, living alone, and living with family); Lifestyle behaviors including physical exercise (yes or no), drinking (yes or no), smoking (yes or no), and sleep duration (less than or equal to 5 h, 6 to 9 h, and great than or equal to 10 h) (40); Chronic conditions including hypertension, diabetes, and stroke/cerebrovascular which were self-reported by respondents. The respondents who answered “yes” when asked whether they suffered the above diseases were classified as hypertension, diabetics, and stroke/cerebrovascular patients, respectively.

### 2.3 Data analysis

Statistical analysis was conducted using the SPSS version 24.0 (IBM Corp, Armonk, NY, USA) for Windows operating system (Microsoft Corp, Redmond, WA, USA). The characteristics of participants with frequency (percentage) were described using descriptive statistics. The χ^2^-test and univariate logistic regression analysis was used to compare the variables of cognitive impairment, including age, marriage status, educational level, place of residence, co-residence status, smoking, drinking, physical exercise, sleep duration, hypertension, diabetes, stroke/cerebrovascular, loneliness, BADL/IADL disability and anxiety. The odds ratios (ORs) were calculated as the exponential coefficient of the logistic model. Pearson’s correlation was used to analyze the correlations between loneliness, ADL, anxiety, and cognitive function. The mediating effects of ADL and anxiety on the association between loneliness and cognitive function were examined using PROCESS macro and multiple linear regression analysis. Besides, 95% bootstrap CI was calculated based on 5,000 bootstrapped samples. A two-sided *p*-value below 0.05 indicated statistical significance.

## 3 Results

### 3.1 Associations between sample characteristics and cognitive function

A total of 10,026 older adults including 4,675 males and 5,351 females aged 60–117 years (mean age: 83.68 ± 11.24 years) were enrolled. In this study, 2,533 (25.3%) participants were defined as cognitive impairment by MMSE. Compared with males, females were 2.468 (OR = 2.468, 95% CI = [2.242, 2.717]) times as likely to develop cognitive impairment. The risk of cognitive impairment of individuals aged 80 and above was 9.931-fold (OR = 9.931, 95% CI = [8.602, 11.465]) that of those aged 60–79. The prevalence of loneliness among older adults was 25.4%. Compared with those who never feel lonely, older people who often feel lonely were associated with a higher risk of cognitive impairment (OR = 1.645, 95% CI = [1.490, 1.816]). Moreover, 19.4% and 61.6% of the participants experienced BADL and IADL disabilities, respectively. Compared to those without BADL and IADL disabilities, people with BADL (OR = 6.721, 95% CI = [6.039, 7.480]) and IADL (OR = 13.806, 95% CI = [11.720, 16.262]) disabilities had a higher risk of cognitive impairment. This analysis showed that 11.2% of the participants suffered from anxiety. Older adults with anxiety (OR = 1.673, 95% CI = [1.466, 1.910]) had a higher risk of cognitive impairment than those without anxiety. See Table 1 for more details.

**Table 1:** Univariate logistic regression analyses of cognitive impairment.

Characteristics		Total (*n* = 10,026)	Cognitive impairment		*P*	OR (95% CI)
			No (*n* = 7,493)	Yes (*n* = 2,533)		
Gender	Male	4,675	3,901 (83.4)	774 (16.6)	–	1.000
	Female	5,351	3,592 (67.1)	1759 (32.9)	<0.001	2.468 (2.242, 2.717)
Age (year)	60 ~ 79	3,931	3,704 (94.2)	227 (5.8)	–	1.000
	≥ 80	6,095	3,789 (62.2)	2,306 (37.8)	<0.001	9.931 (8.602, 11.465)
Marriage status	Have a spouse	4,597	4,089 (88.9)	508 (11.1)	–	1.000
	No spouse	5,429	3,404 (62.7)	2025 (37.3)	<0.001	4.788 (4.301, 5.331)
Educational level	Illiterate	4,561	2,664 (58.4)	1897 (41.6)	–	1.000
	Non-illiterate	5,465	4,829 (88.4)	636 (11.6)	<0.001	0.185 (0.167, 0.205)
Place of residence	City and town	5,590	4,269 (76.4)	1,321 (23.6)	–	1.000
	Rural	4,436	3,224 (72.7)	1,212 (27.3)	<0.001	1.215 (1.110, 1.330)
Co-residence status	Living with family	8,074	6,060 (75.1)	2014 (24.9)	–	1.000
	Living alone	1,635	1,240 (75.8)	395 (24.2)	0.503	0.958 (0.847, 1.085)
	Living in nursing home	317	193 (60.9)	124 (39.1)	<0.001	1.933 (1.534, 2.436)
Smoking	No	8,413	6,166 (73.3)	2,247 (26.7)	–	1.000
	Yes	1,613	1,327 (82.3)	286 (17.7)	<0.001	0.591 (0.516, 0.678)
Drinking	No	8,436	6,160 (73.0)	2,276 (27.0)	–	1.000
	Yes	1,590	1,333 (83.8)	257 (16.2)	<0.001	0.522 (0.453, 0.601)
Physical exercise	No	6,668	4,588 (68.8)	2080 (31.2)	–	1.000
	Yes	3,358	2,905 (86.5)	453 (13.5)	<0.001	0.344 (0.308, 0.385)
Sleep duration	≤ 5 h	1869	1,361 (72.8)	508 (27.2)	–	1.000
	6 ~ 9 h	6,368	5,062 (79.5)	1,306 (20.5)	<0.001	0.691 (0.614, 0.778)
	≥ 10 h	1789	1,070 (59.8)	719 (40.2)	<0.001	1.800 (1.567, 2.069)
Hypertension	No	5,792	4,142 (71.5)	1,650 (28.5)	–	1.000
	Yes	4,234	3,351 (79.1)	883 (20.9)	<0.001	0.661 (0.602, 0.726)
Diabetes	No	9,024	6,650 (73.7)	2,374 (26.3)	–	1.000
	Yes	1,002	843 (84.1)	159 (15.9)	<0.001	0.528 (0.443, 0.630)
Stroke/Cerebrovascular	No	8,936	6,691 (74.9)	2,245 (25.1)	–	1.000
	Yes	1,090	802 (73.6)	288 (26.4)	0.352	1.070 (0.928, 1.235)
Loneliness	Not lonely	7,480	5,778 (77.2)	1702 (22.8)	–	1.000
	Lonely	2,546	1715 (67.4)	831 (32.6)	<0.001	1.645 (1.490, 1.816)
BADL disability	No	8,077	6,682 (82.7)	1,395 (17.3)	–	1.000
	Yes	1949	811 (41.6)	1,138 (58.4)	<0.001	6.721 (6.039, 7.480)
IADL disability	No	3,852	3,686 (95.7)	166 (4.3)	–	1.000
	Yes	6,174	3,087 (61.7)	2,367 (38.3)	<0.001	13.806 (11.720, 16.262)
Anxiety	No	8,904	6,760 (75.9)	2,144 (24.1)	–	1.000
	Yes	1,122	733 (65.3)	389 (34.7)	<0.001	1.673 (1.466, 1.910)
Total		10,026	7,493 (74.7)	2,533 (25.3)		

Categorical variables are presented as the frequencies and percentages. *p*-values were calculated by χ^2^-tests. ORs, abbreviation of odds ratios, and were calculated as the exponentiated coefficient from logistic models. 95% CI, abbreviation of 95% confidence intervals.

### 3.2 Analysis of correlations

Table 2 shows the means, standard deviations, and bivariate correlations of all study variables. Loneliness, ADL, and anxiety had significantly negative correlations with cognitive function, with a correlation coefficient of −0.144, −0.615 and −0.105, respectively. Loneliness was positively related to ADL (*r* = 0.137, *p* < 0.001) and anxiety (*r* = 0.338, *p* < 0.001), while ADL was also positively related to anxiety (*r* = 0.104, *p* < 0.001).

**Table 2:** Correlations among study variables.

Variables	1	2	3	4
1. Loneliness	1			
2. ADL	0.137^***^	1		
3. Anxiety	0.338^***^	0.104^***^	1	
4. Cognitive function	−0.144^***^	−0.615^***^	−0.105^***^	1

^***^p < 0.001.

### 3.3 Mediation effect evaluation

Based on the mediation effect test method proposed by MacKinnon (41), the mediating effects of ADL and anxiety on the relationship between loneliness and cognitive function in Chinese older adults were examined. Table 3 shows the results adjusted for gender, age, marriage status, educational level, place of residence, co-residence, smoking, drinking, physical exercise, sleep duration, hypertension, diabetes, and stroke/cerebrovascular. First, the Model 1 was significant (*F* = 250.338, *p* < 0.001, *R^2^* = 0.259) with a negative relationship between loneliness and cognitive function (*B* = −0.387, *p* < 0.001). Second, Model 2 (*F* = 353.480, *p* < 0.001, *R^2^* = 0.331) and Model 3 (*F* = 120.097, *p* < 0.001, *R^2^* = 0.153) were both significant. Loneliness and ADL were significantly positively correlated (*B* = 0.265, *p* < 0.01), while ADL was also significantly positively correlated with anxiety (*B* = 0.041, *p* < 0.001). Finally, Model 4 was also significant (*F* = 476.951, *p* < 0.001, *R^2^* = 0.433). When the variable of loneliness, ADL and anxiety were simultaneously included in the regression equation to predict cognitive function, loneliness was still related to cognitive function (*B* = −0.199, *p* < 0.001), ADL was negatively related to cognitive function (*B* = −0.437, *p* < 0.001), and anxiety was also negatively related to cognitive function (*B* = 0.078, *p* < 0.001).

**Table 3:** Multiple linear regression analysis of loneliness on cognitive function.

Independent variables	Model 1 (Cognitive function)		Model 2 (ADL)		Model 3 (Anxiety)		Model 4 (Cognitive function)	
	*B*	*t*	*B*	*t*	*B*	*t*	*B*	*t*
Loneliness	−0.387	−6.766^***^	0.265	4.237^**^	0.903	33.852^***^	−0.199	−3.766^***^
ADL					0.041	9.579^***^	−0.437	−54.482^***^
Anxiety							−0.078	−4.180^***^
*R^2^*	0.259		0.331		0.153		0.433	
*F*	250.338^***^		353.480^***^		120.097^***^		476.951^***^	

The results adjusted for gender, age (year), marriage status, educational level, place of residence, co-residence status, smoking, drinking, physical exercise, sleep duration, hypertension, diabetes, and stroke/cerebrovascular. ^***^p < 0.001, ^**^p < 0.01.

**Figure 3:**
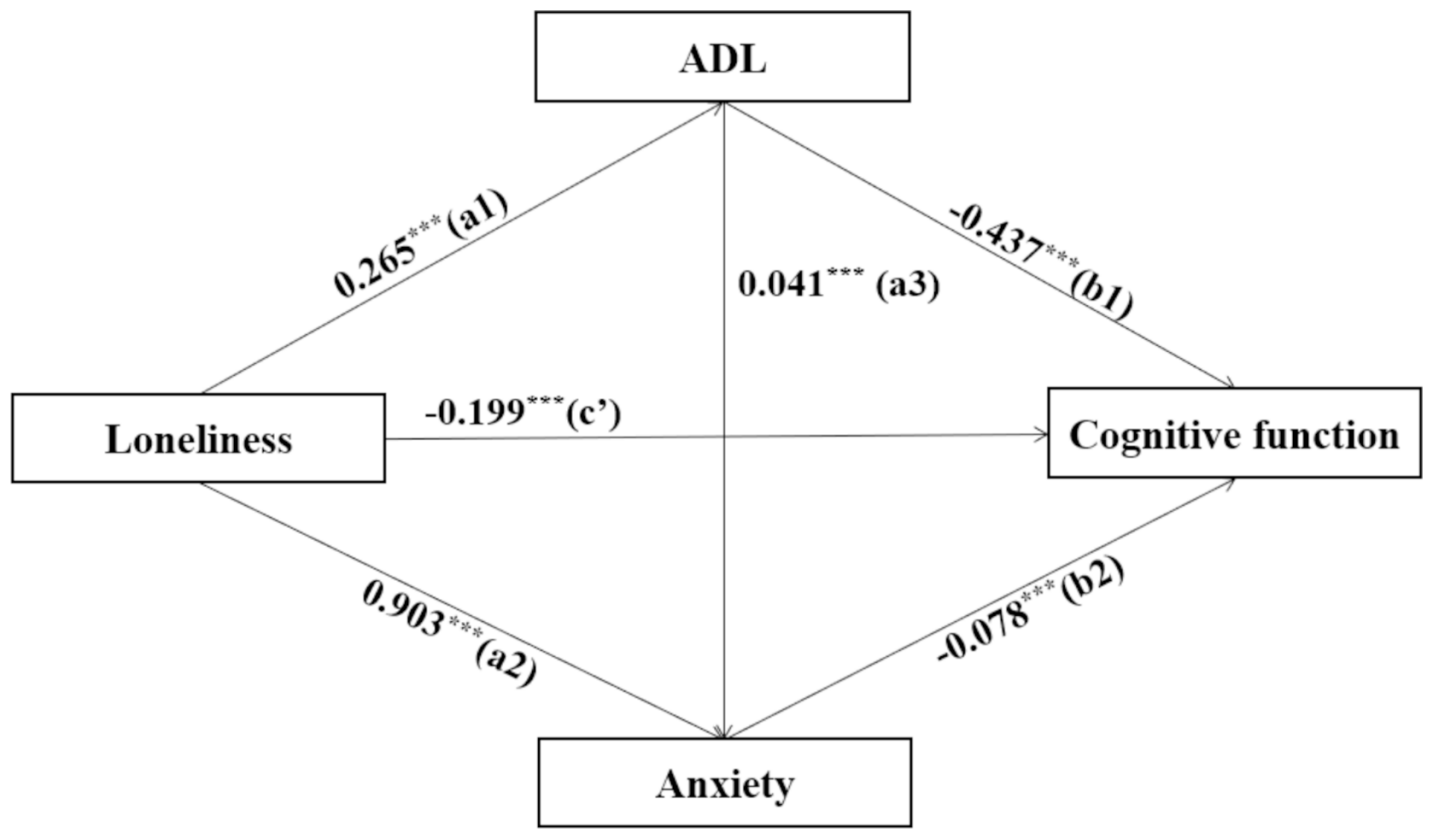
The serial mediation effects of ADL and anxiety on the association between loneliness and cognitive function.

Based on the PROCESS macro (Model 6) of SPSS, the bootstrap method was used to confirm the serial multiple mediation effect of loneliness on cognitive function through ADL and anxiety. The results found that the three mediating effects were significant (Table 4 and Figure 3). The 95% CI was calculated based on 5,000 bootstrap resampling, which showed that the direct effect of loneliness on cognitive function was equal to −0.1991 (95% CI = [−0.3027, −0.0955]; excluding 0), with a total effect value of −0.3866 (95% CI = [−0.4987, −0.2746]; excluding 0) and a total indirect effect value of −0.1875 (95% CI = [−0.2579, −0.1158]; excluding 0). Among them, the indirect 1 effect of loneliness on cognitive function via ADL was significant (95% CI = [−0.1735, −0.0582]), which represented a total effect of 30.0%. The indirect 2 effect of loneliness on cognitive function via anxiety was significant (95% CI = [−0.1092, −0.0323]), which represented a total effect of 18.3%. The indirect 3 effect of loneliness on cognitive function via ADL and anxiety was also significant (95% CI = [−0.0016, −0.0003]). Therefore, the roles of ADL and anxiety in partially mediating the relationship between loneliness and cognitive function were confirmed.

**Table 4:** Hypothesized serial mediation model of ADL and anxiety between loneliness and cognitive function.

Pathway	Effect	SE	Boot LLCI	Boot ULCI	Share of the total effect, %
Total effect (c)	−0.3866	0.0571	−0.4987	−0.2746	—
Direct effect (c’)	−0.1991	0.0529	−0.3027	−0.0955	51.5
a1	0.2651	0.0626	0.1425	0.3878	
a2	0.9031	0.0267	0.8508	0.9553	
a3	0.0408	0.0043	0.0324	0.0491	
b1	−0.4372	0.0080	−0.4530	−0.4215	
b2	−0.0784	0.0188	−0.1152	−0.0416	
Indirect effects					
Total indirect effects	−0.1875	0.0360	−0.2579	−0.1158	48.5
Indirect 1	−0.1159	0.0295	−0.1735	−0.0582	30.0
Indirect 2	−0.0708	0.0198	−0.1092	−0.0323	18.3
Indirect 3	−0.0008	0.0003	−0.0016	−0.0003	0.2

Indirect 1, loneliness → ADL → cognitive function; Indirect 2, loneliness → anxiety → cognitive function; Indirect 3, loneliness → ADL → anxiety → cognitive function. Boot LLCI, bootstrapping lower limit confidence interval; Boot ULCI, bootstrapping upper limit confidence interval; SE, standard error. The results adjusted for gender, age (year), marriage status, educational level, place of residence, co-residence status, smoking, drinking, physical exercise, sleep duration, hypertension, diabetes, and stroke/cerebrovascular.

## 4 Discussion

The present study investigated the status of cognitive function and the associations of loneliness, ADL, and anxiety with cognitive function among older adults in China. It was found that (1) more than a quarter of older adults suffered from cognitive impairment, (2) loneliness, ADL, and anxiety had negative correlations with cognitive function, and (3) ADL and anxiety played multiple mediating roles in the relationship between loneliness and cognitive function. These results highlight the critical importance of targeting and improving the “loneliness-ADL-anxiety” pathway as a preventive strategy for effectively reducing the risk of cognitive impairment among older adults in China. This may provide important scientific support for the development of public health policy and address the challenges posed by the aging population worldwide.

In this study, the prevalence of cognitive impairment in older adults was 25.3%, which was higher than that in India (13.7%) (42) and Japan (13.3%) (43). These differences may be partly attributed to variations in sample characteristics, diagnostic criteria, and screening tools. Cognitive impairment is a complex mental and psychological condition that seriously endangers the health of older adults. In China, with rapid population aging, the number of older adults with cognitive impairment will continue to increase. The social pension and medical burden will be also aggravated by the influence on the independence and autonomy of older people’s survival and adaptation. Hence, it is essential to closely monitor the cognitive function of older adults and use early screening tests.

This study confirmed that loneliness had a direct negative association with cognitive function, meaning that higher levels of loneliness indicate a greater risk of cognitive impairment. A longitudinal study indicated that persistent loneliness was strongly associated with accelerated cognitive decline (8). In the present study, the prevalence of loneliness among older adults was 25.4% (2,546/10,026), and that of cognitive impairment in the lonely population was 32.6% (831/2,546). The evolutionary theory of loneliness suggests that loneliness as a stressor may lead to dysregulation in multiple physiological systems. Loneliness may reduce neural reserve by reducing dendritic arborization in the hippocampus and prefrontal cortex after triggering long-term activation of the hypothalamus–pituitary–adrenal (HPA) axis, which leads to a decrease in learning and memory (44). Also, loneliness may cause proinflammatory responses, which in turn leads to cognitive impairment (45, 46). A recent study by Moretti et al. (47) similarly found a negative association between loneliness and cognitive function in patients with major neurocognitive disorders, reinforcing our conclusion. Moreover, Rodrigues et al. (28) reported in a meta-analysis that loneliness increases the risk of Alzheimer’s disease and suicidal behaviors, underscoring its broad adverse effects on cognitive and mental health.

In this study, the mediating effect of loneliness on cognitive function via ADL represented the highest proportion (30.0%) of indirect effects. More severe loneliness indicates worse ADL and cognitive levels, which is consistent with hypothesis H1. Loneliness contributes to less engagement in fewer health-promoting behaviors (e.g., physical inactivity, malnutrition) (48), which in turn results in declined physical function. It is evidenced that chronic loneliness is an independent risk factor for new-onset ADL disability in middle-aged and older adults (49). At the same time, the level of ADL affects the occurrence of cognitive decline among older adults (50). Impaired ADL may reduce the opportunity for older adults to participate in daily activities (e.g., shopping, walking around the community) and prevent them from performing social roles. This leads to reduced participation in social interaction, which is an important way for them to obtain information and social support. The amount of effective stimulation required for the human brain can be reduced by these effects, which accelerates the decline of cognitive function.

This study found that loneliness could affect cognitive function not only directly but also partially via the mediating effect of anxiety among older adults. Wang et al. observed significant mediating effects on the relationship between loneliness and cognitive function through anxiety and sleep disturbances (24). However, one study is inconsistent with this result. Power et al. found that anxiety failed to mediate the relationship between loneliness and cognitive decline (51). Both emotional and social loneliness have been reported to be significantly related to psychological health (52). With retirement and declined physical function, the social function of older adults is reduced, coupled with the long-term absence of children and the death of spouses or friends, which may lead to reduced social activities. The spirits of older adults can be easily dampened by feeling lonely and not being accepted by the outside world. Results from a population-based study presented a positive association of loneliness with anxiety (53). Anxiety can increase the risk of cognitive impairment and interfere with cognitive function by shifting attention and focusing on information related to fear and threat. Campos et al. indicated that those with anxiety symptoms had a 64% higher prevalence of cognitive impairment (54). Therefore, this study indicated that loneliness may disrupt emotion and then decrease cognitive levels.

The findings indicate that loneliness affects ADL and then ADL affects cognitive function through an indirect pathway that partially mediates the effects of anxiety. Older adults who experience loneliness may lack the social support and motivation necessary to perform routine tasks, thereby exacerbating functional limitations. Poorer ADL indicates that individuals have a lower ability to independently engage in fundamental and instrumental daily activities, which leads to feelings of worthlessness or hopelessness due to the loss of independent identity. Moreover, the lack of self-care ability will significantly reduce the frequency of interpersonal communication, participation in social activities and the ability to overcome negative emotions effectively, leading to the psychological environment of anxiety development. Difficulty in ADL is a potential predictor of high-risk anxiety (55). Anxiety may act as a cognitive load, consuming mental resources that could otherwise be allocated to performing complex tasks or retaining new information. Anxiety among older adults often persists throughout life and affects language memory, which results in the decline of cognitive function (56, 57). This study showed that older adults who experience loneliness may have a decline in ADL, and excessive dependence on others in life may damage their mental health and even cause anxiety. Consequently, this leads to the development of cognitive impairment.

The above results have significant implications for public health initiatives and clinical practices. Targeted interventions should address loneliness, ADL, and anxiety to improve cognitive function among older adults. First, community-based social engagement programs, such as senior centers, group activities, and intergenerational interactions, can mitigate loneliness and promote psychological stimulation. The provision of accessible mental health services, such as counseling or anxiety management workshops, can further support cognitive health. Second, tailored physical and occupational therapy programs should be implemented to enhance ADL independence, with a focus on improving mobility and self-care. At the family level, caregivers should be educated about the importance of emotional support and encouraged to maintain regular communication with older adults. Families can also assist in creating a structured routine that supports ADL independence while ensuring safety. Third, at the individual level, older adults should be encouraged to participate in cognitive training, such as puzzles or memory games, and to engage in regular physical activities to maintain both mental and physical health. Additionally, mindfulness practices and stress-reduction techniques can help control anxiety. By fostering collaboration at these levels, a supportive environment can be created to enhance cognitive function and overall well-being in older adults.

### 4.1 Limitations

This study also has some limitations. First, anxiety variables are newly included in the CLHLS 2018 database. Due to the limitations of the database, a longitudinal research design cannot be adopted and the causal relationship of the obtained associations cannot be determined, which may affect the rigor of the results. In particular, while the mediation model assumed that loneliness contributes to poorer cognitive function, the reverse pathway is equally plausible given the cross-sectional design. That is, cognitive decline may lead to reduced social engagement and increased loneliness. Second, the data on loneliness, ADL, anxiety, and cognitive function come from self-reports and may induce several recall biases. In addition, loneliness was measured using a single-item question, which does not distinguish between different dimensions of loneliness, emotional versus social loneliness. In the future, more objective assessment methods, including multidimensional loneliness scales, should be adopted to enhance the validity of the findings and the reliability of the data. Finally, although this study controlled for several important confounding factors, it did not consider other factors that could affect cognitive function.

## 5 Conclusion

This study investigated the mediating effects of ADL and anxiety on the relationship between loneliness and cognitive function among older adults. The results revealed a significant chain-mediating effect, which suggests that loneliness has an indirect impact on cognitive function by independently and accumulatively affecting ADL and anxiety. This finding provides valuable insights into the complex mechanisms of cognitive decline in older adults and highlights the importance of addressing both psychological and functional factors in interventions designed to protect cognitive health. Addressing these factors holistically may provide a promising pathway for improving the quality of life and cognitive health of older adults.

## Data Availability

Publicly available datasets were analyzed in this study. This data can be found at: https://opendata.pku.edu.cn/dataset.xhtml?persistentId=doi:10.18170/DVN/WBO7LK&version=2.0.
